# Serial Recall Order and Semantic Features of Category Fluency Words to Study Semantic Memory in Normal Ageing

**DOI:** 10.3389/fnagi.2021.678588

**Published:** 2021-08-03

**Authors:** Matteo De Marco, Daniel J. Blackburn, Annalena Venneri

**Affiliations:** ^1^Department of Life Sciences, Brunel University London, London, United Kingdom; ^2^Department of Neuroscience, The University of Sheffield, Sheffield, United Kingdom

**Keywords:** semantic memory, efficiency, centrality, hippocampus, Alzheimer’s disease, pre-clinical

## Abstract

**Background:** Category Fluency Test (CFT) is a common measure of semantic memory (SM). Test performance, however, is also influenced by other cognitive functions. We here propose a scoring procedure that quantifies the correlation between the serial recall order (SRO) of words retrieved during the CFT and a number of linguistic features, to obtain purer SM measures. To put this methodology to the test, we addressed a proof-of-concept hypothesis whereby, in alignment with the literature, older adults would show better SM.

**Methods:** Ninety participants (45 aged 18–21 years; 45 aged 70–81 years) with normal neurological and cognitive functioning completed a 1-min CFT. SRO was scored as an ordinal variable incrementing by one unit for each valid entry. Each word was also scored for 16 additional linguistic features. Participant-specific normalised correlation coefficients were calculated between SRO and each feature and were analysed with group comparisons and graph theory.

**Results:** Younger adults showed more negative correlations between SRO and “valence” (a feature of words pleasantness). This was driven by the first five words generated. When analysed with graph theory, SRO had significantly higher degree and lower betweenness centrality among older adults.

**Conclusion:** In older adults, SM relies significantly less on pleasantness of entries typically retrieved without semantic control. Moreover, graph-theory metrics indicated better optimised links between SRO and linguistic features in this group. These findings are aligned with the principle whereby SM processes tend to solidify with ageing. Although additional work is needed in support of an SRO-based item-level scoring procedure of CFT performance, these initial findings suggest that this methodology could be of help in characterising SM in a purer form.

## Introduction

Beyond its use in linguistics and neurology as a term to indicate the flow of language, *verbal fluency* identifies a cognitive ability that supports retrieval from memory (Patterson, 2011) and that is commonly used to assess semantic memory (SM). Measures of SM are particularly important to the study of cognitive ageing. Findings from large cohorts of asymptomatic adults followed up longitudinally, in fact, have revealed that performance on a major SM test, the “Category Fluency Test” (CFT) (inclusive of its analogues, e.g., the “Isaacs Set Test”), is among the earliest predictors of future progression to Alzheimer’s disease (Amieva et al., 2008; Payton et al., 2020). Conversely, a large body of evidence indicates that SM tends to be largely preserved and even improve with healthy ageing (Nyberg et al., 1996, 2003; Park et al., 2002; Verhaeghen, 2003; Rönnlund et al., 2005; Small et al., 2011). Although a decrease in performance has been frequently reported in older adults on the CFT, this is thought, however, to be accounted for by decline of other supportive abilities such as executive functioning and processing speed (Spaan, 2015; Aita et al., 2019; Gonzalez-Burgos et al., 2019). In this respect, although CFT performance is widely regarded, for all intents and purposes, as an index of SM (Venneri et al., 2016, 2018), a number of studies have included it as part of the assessment of executive functioning (Rende et al., 2002; Gibbons et al., 2012). Executive abilities, in fact, go further than providing simple external facilitatory resources to task engagement. SM, in fact, relies on an intrinsic executive component, “semantic control”, that supports manipulation of semantic content to facilitate retrieval (Lambon Ralph et al., 2017). In addition, performance on this test is also influenced by other functions such as processing speed (Elgamal et al., 2011) and episodic memory (Greenberg et al., 2009). Furthermore, clinicians often consider CFT scores as reflecting expressive language abilities, since disrupted SM retrieval affects linguistic production and may interfere with effective communication. Although this evidence clearly indicates that the CFT has been thoroughly investigated in relation to a variety of cognitive functions, no conclusive framework has yet been outlined and no study has quantified the contribution of each distinct function to test performance in the context of ageing.

There is a clinical interest in assessing SM in the most accurate possible way. The latest clinical diagnostic guidelines for Alzheimer’s disease discourage the use of available biomarkers as the sole diagnostic features at the pre-clinical stage (Dubois et al., 2021). It is thus of central importance to explore alternative methodological routes that can help identify subtle changes indicative of early stage neurodegeneration. In this respect, SM may play a crucial role (Venneri et al., 2016). Alternative methodologies have been studied to overcome the multi-componential element that characterises the construct validity of standard CFT scoring, to obtain “purer” measures of SM. A large number of studies have investigated the semantic properties of words generated during performance on CFTs, such as “age of acquisition,” “typicality,” and “frequency,” i.e., “item-level features” (Forbes-McKay et al., 2005; Biundo et al., 2011; Venneri et al., 2011; Vita et al., 2014; Quaranta et al., 2016; Wakefield et al., 2018; Vonk et al., 2019a,b; Taler et al., 2020), under the assumption that the ability to generate less frequent, less typical and later acquired words would reflect efficient semantic processing (Murray and Forster, 2004; Steyvers and Tenenbaum, 2005; Plant et al., 2011). Other studies have focussed on the semantic relationships between words (e.g., Goñi et al., 2011; Pakhomov et al., 2012; Bertola et al., 2014; Quaranta et al., 2019), on the assumption that the sequence of words could be indicative of the integrity of the underlying semantic-processing system.

**FIGURE 1:**
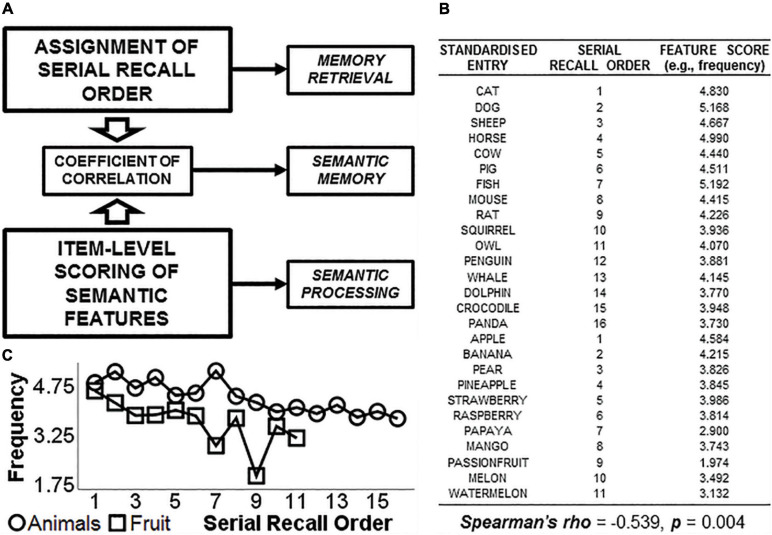
Graphical representation of the principle at the basis of the study. While the serial order of recall is a property of memory retrieval, features such as word “frequency,” “typicality,” or “age of acquisition” are linked to semantic processing. The calculation of a coefficient of correlation between these two variables would produce an index that can inform how retrieval from memory is associated with semantic “difficulty” of words, and thus provide a theoretically valid measure of semantic memory **(A)**. On the right, a practical example of feature-to-feature correlation between “serial recall order” and “frequency” **(B)**. This is illustrated in the bottom left corner **(C)**.

In this exploratory study we combined the principles of item-level and sequence-related properties to test a novel approach to CFT scoring that combines aspects of semantic processing with a property of memory retrieval. Specifically, we focussed on the positional order with which words are retrieved from memory during the process of word generation required by the test (i.e., first word recalled, second word recalled, third word recalled…), the serial recall order (SRO). The SRO score (Figure 1A) is operationalisable as an ordinal variable ranging from 1 (first word generated) and incrementing by one unit up to *n* (*n*th word generated). Typically, words with higher frequency of use in a given language are generated during the first temporal segment of the minute trial (Crowe, 1998), suggesting a negative association between SRO and frequency (i.e., as the positional order increases, less frequent words are generated). This indicates that, as the category is explored in greater depth as part of the test, words generated toward the end of the trial tend to become “more difficult” exemplars, at least as far as frequency is concerned (i.e., Figures 1B,C). Moreover, a recent study found that, as categories are explored, *more original* entries tend to be generated, i.e., words given by less than 5% of the target cohort (Murphy and Castel, 2020).

To capture the association between SRO (a property of memory retrieval) and word features such as frequency, typicality or age of acquisition (properties of semantic processing), we calculated a series of subject-specific coefficients of correlation that quantify the trend shown by a participant’s word production becoming “more difficult” as more entries are generated. We assumed that the idea of “getting more difficult” would translate into decreasing word frequency, decreasing typicality, increasing age of acquisition and further increases or decreases in a number of semantic properties (described in section “CFT–Scoring Procedures”) linked to the target category (e.g., “animals”). We propose that these correlations capture the interplay of memory retrieval and semantic processing, and that aspects of SM are expressed by this interplay (i.e., as illustrated schematically in Figure 1). Supporting functions such as processing speed or executive functioning are well known to have a significant impact on word count (Rende et al., 2002; Elgamal et al., 2011; Gibbons et al., 2012). As long as correlations are stable (i.e., based on a sufficiently large sample size), however, they can be equally calculated regardless of the exact number of entries. Based on this, we formulated a first, methodological hypothesis: supporting functions will show a statistical effect on the number of valid words generated via semantic control and via control of retrieval processes, but not on the interplay between SRO and semantic features.

We then relied on this framework to test a second, experimental hypothesis designed *ad hoc* and meant to lay the thematic foundations for this line of research. To this end, we analysed retrospectively the CFT performance of 45 younger adults and 45 older adults. Since, as highlighted by the literature, SM tends to consolidate with ageing (Nyberg et al., 1996, 2003; Park et al., 2002; Verhaeghen, 2003; Rönnlund et al., 2005; Small et al., 2011), we expected that this set of correlation coefficients would show significant group differences indicating higher levels of semantic organisational structure among older adults. Older adults would thus show significantly stronger correlations in the same direction (i.e., positive or negative) as that shown by younger adults (e.g., among others, a significantly stronger negative correlation between SRO and typicality and between SRO and frequency, and a significantly stronger positive correlation between SRO and age of acquisition would be expected). To address this hypothesis, we tested for group differences via the direct comparison of standardised coefficients of correlation and via the exploratory analysis of nodal properties of SRO, as informed by graph theory.

## Materials and Methods

### Participants

This study is based on the secondary analysis of datasets collected on cognitively normal volunteers. These had been originally recruited as part of a large cohort for the purpose of collecting in-house normative data for neuropsychological test scores, to be used as numerical reference to aid profiling of neurological patients in tertiary care. Two distinct age groups were targeted in this study (Table 1): volunteers between 18 and 21 years of age (henceforth, “younger adults”) and between 70 and 81 (henceforth, “older adults”). The choice of comparing two distant age groups was guided by normative studies of CFT [see (Woods et al., 2016) for a study carried out in English native speakers]: these studies show that CFT performance across the entire adulthood can be accounted for by a single normative model.

**TABLE 1:** Demographic and neuropsychological description of the sample.

Variable	Younger Adults	Older Adults	*p*
***Demographic Indices***			
Age (years)	19.09 (1.10)	73.89 (3.08)	<0.001
Education (years)	14.00 (1.51)	13.89 (3.04)	0.827
Sex (f/m)	26/19	28/17	0.667
Mini-Mental State Examination	29 (2)	29 (2)	0.987
***Neuropsychological Assessment–Non-normally Distributed Tests***			
Confrontation Naming Test	18 (2)	20 (1)	<0.001
Paired Associated Learning Test	19 (5)	15 (6)	<0.001
Pyramids and Palm Trees Test	49 (3)	51 (2)	<0.001
Rey-Osterrieth Complex Figure Test–Copy	35 (3)	34 (5)	0.058
Rey-Osterrieth Complex Figure Test–Recall	22 (7.275)	16 (9.5)	<0.001
Digit Span Test–Forward	7 (2)	7 (3)	0.983
Digit Span Test–Backward	5 (2)	5 (3)	0.244
Raven Coloured Progressive Matrices	33 (4)	33 (3)	0.381
Digit Cancellation Test	56 (4)	54 (7)	0.006
Visuoconstructive Apraxia Test	14 (0)	13 (2)	<0.001
Stroop Test–Time Interference	10.3 (6.07)	21.5 (13.1)	<0.001
Stroop Test–Error Interference	0 (0)	0 (0)	0.900
Token Test	34 (1.5)	35 (2)	0.122
***Neuropsychological Assessment–Normally Distributed Tests***			
WAIS–Similarities Test	20.31 (4.46)	22.56 (6.60)	0.063
Letter Fluency Test	39.02 (10.32)	45.56 (15.53)	0.021
***Category Fluency Test–Normally Distributed Indices***			
Test score: Two Categories	33.80 (6.65)	33.69 (7.02)	0.939
Category: Animals	19.60 (4.47)	18.67 (4.62)	0.333
Category: Fruits	14.20 (3.27)	15.02 (4.45)	0.321
***Category Fluency Test–Non-normally Distributed Indices***			
Intrusions	0 (0)	0 (0)	0.746
Perseverations	2 (3)	3 (3)	0.064

*“Age” and “Education” are typically normally distributed and are thus reported as means and standard deviations and analysed with *t*-tests.*

*“Sex” is indicated as frequency ratios and was analysed with a *chi-square* test. Scores on the Mini-Mental State Examination were not normally distributed and are thus indicated as medians and interquartile ranges and analysed with a Mann–Whitney *U* test. Neuropsychological indices were also split into normally and non-normally distributed and reported as appropriate. Scores included in this table reflect uncorrected neuropsychological data.*

A screening questionnaire was completed by each participant prior to recruitment to rule out exclusion criteria of medical or psychological nature that might otherwise have had an impact on neurological and cognitive profiles. These included: diagnostic entities or clinical signs mechanistically linked to psychological health such as neurological conditions or symptoms (e.g., childhood seizures, autistic spectrum, head injury or concussion, history of transient ischaemic attacks, cerebrovascular disease, peripheral neuropathy) cardiovascular conditions of relevance (e.g., atrial fibrillation, uncontrolled diabetes, hypertension or hypercholesterolemia, sick-sinus syndrome, obstructive sleep apnoea, chronic obstructive pulmonary disease, history of cardiovascular surgery), metabolic dysfunctions (e.g., folate/vitamin B12 malabsorption, abnormal levels of thyroid-stimulating hormone, lactose/gluten intolerance), ongoing pharmacological treatment with psychotropic or experimental medications, or with molecules with known toxic effects on internal organs, substance abuse, learning disabilities and presence of behavioural symptoms suggestive of underlying psychological dysfunction or difficulties (e.g., addiction, chronic anxiety/depression/apathy, mood or personality disorders, attention deficit hyperactivity disorder). Each volunteer was invited to the Department of Neuroscience at the University of Sheffield (United Kingdom) and completed a battery of neuropsychological tests. No participant had subjective cognitive complaints. Of the two groups, particular care was taken to evaluate diagnostic statuses in the group of older adults, since in this age range prevalence of cognitive impairment is estimated to range between 5% and 40% (Pais et al., 2020). To assess their cognitive profile the diagnostic labelling consensus proposed by the American Academy of Clinical Neuropsychology was followed, whereby performance above the expected 24th percentile is considered within normal limits (Guilmette et al., 2020). We thus used the entire cohort of ≥70 year-old adults (*n* = 75) from which the study group of older adults had been extracted, to define numerical cut-offs corresponding to the 24th, 8th, and 2nd percentile for each test score. This was carried out to categorise performance into one of the following four labels: “*score within normal limits*”, “*low average score*”, “*below average score*”, and “*exceptionally low score*” (Guilmette et al., 2020). For clinical interpretational purposes, we also relied on the principles outlined by Axelrod and Wall (2007) and by Binder et al. (2009), according to which a proportion of scores not within normal limits should be expected when a battery of tests is administered to healthy controls.

All participants provided their written informed consent prior to study inclusion. All procedures were carried out in compliance with the Declaration of Helsinki. The study was approved by the regional ethics committee of Yorkshire and Humber, reference number 05/Q1104/129.

### CFT–Scoring Procedures

The “classic” 1-min version of the test was administered orally. Three categories were used: cities, animals and fruits (in this order). For the purposes of this study, only animals and fruits were analysed, since “cities” is a category based on the recall of proper nouns for which no linguistic ratings are available. Sub-scores on these two categories were modelled to evaluate cross-category consistency. Linear regression models were run to predict the number of correct “fruits” entries using the number of correct “animals” entries as predictor. This was carried out in the entire cohort and, separately, for each age group.

Each test performance was carefully reviewed and entries were scored as correct if they belonged to the target category (i.e., were not “intrusions”) and if they were not “perseverations,” (e.g., a repetition, a subordinate/superordinate to a word already produced such as “*ape*” and “*gorilla*,” or the same entity in a different context such as “*grape*” and “*raisin*,” or “*sheep*” and “*lamb*”). For a detailed description of these rules, please refer to the Supplementary Material. To ensure consistency in the scoring procedures across all 90 participants, a standardised form was defined for each entry that had been generated in multiple ways (e.g., “*kiwi*” and “*kiwi fruit*,” or “*hippo*” and “*hippopotamus*”). Please consult the Supplementary Material for more details on standardised entries. All intrusions and perseverations were discarded. *Post hoc* analyses were, however, run on these data.

Each word was scored based on 17 item-level semantic and non-semantic descriptors: *typicality*, *age of acquisition*, *concreteness*, *frequency*, *prevalence*, *recognition time*, *valence*, *arousal*, *dominance*, *body-object interaction*, *graphemes count*, *syllables count*, *consonant/vowel quantity ratio*, *phonological complexity*, *SRO*, *in-list orthographic Levenshtein distance*, and *dictionary orthographic Levenshtein distance*. A description of these features (inclusive of examples) and the references from which linguistic ratings were obtained (Rosch, 1975; Murray and Forster, 2004; Yarkoni et al., 2008; Hargreaves et al., 2012; Kuperman et al., 2012; Warriner et al., 2013; Brysbaert et al., 2014, 2019; van Heuven et al., 2014; Dufau et al., 2015; Riley and Thompson, 2015; Räling et al., 2016; Pexman et al., 2019; Sohrabi, 2019; Mandera et al., 2020) are listed in Table 2.

**TABLE 2:** Description, inclusive of examples, of all 17 features included in this study.

Feature	Description of Feature	Example (Category: Animals)	Reference for Normative Data
**Semantic Features**			
*Typicality*	This feature reflects the “prototype approach” of conceptual organisation, which posits that semantic categories are organised based on an internal structure (Rosch, 1975) and that each word is characterised by a degree of semantic relatedness with other words of that category (Räling et al., 2016). Within this structure, some members of the category are more typical exemplars and are recalled more promptly.	OSTRICH: lower typicality (score = **1.36**); MOOSE: higher typicality (score = **6.42**).	In-house normative data were applied to score this feature: a group of volunteers had been asked to rate how representative a word was of its own category, assigning a score from 1 (least typical) to 7 (most typical).
*Age of Acquisition*	Words acquired earlier in life have had time and opportunity to “sediment” more profoundly in the semantic system and solidify connections with other words than words acquired later in life. As a result, they are processed more rapidly and are more resistant to neural dysfunction (Sohrabi, 2019).	DUCK: earlier age of acquisition (estimated average: **3.53** years); CONDOR: later age of acquisition (estimated average: **13.08** years).	Kuperman et al., 2012
*Concreteness*	This feature (expressed as a number ranging from 1 to 5) was included as a control descriptor under the assumption that, to some extent, all animal and fruit words would be equally concrete. Although skewed towards a score of 5, perceived concreteness of animal words was, possibly, in part “attenuated” by alternative meanings (e.g., MOLE, MANDARIN, to blow a RASPBERRY, etc.).	THRUSH: lower concreteness (score = **3.92**); WALRUS: maximum concreteness (score = **5.00**).	Brysbaert et al., 2014
*Frequency*	The frequency upon which each word appears in a certain language is significantly linked to how difficult/easy it is to access it from semantic memory (Murray and Forster, 2004). A 1-to-7 scale was used to quantify this feature.	MANATEE: lower frequency (score = **2.08**); FISH: higher frequency (score = **5.19**).	The SUBTLEX database for British English (van Heuven et al., 2014).
*Prevalence*	This feature (expressed as *z*-converted percentages) indicates the proportion of people in a population who report they know the word in question, and captures aspects of word difficulty different from those tagged by other indices such as frequency or age of acquisition (Brysbaert et al., 2019).	DORMOUSE: lower prevalence (score = **0.31**); SLOTH: higher prevalence (score = **2.58**).	The English Crowdsourcing Project, an internet-based initiative in which native English speakers were asked to indicate whether they knew a certain word or not (Mandera et al., 2020).
*Recognition Time*	This feature reflects the *z*-converted response time with which study participants indicated that they knew a specific word (Mandera et al., 2020). Recognition time is complementary to prevalence and provides fine-grained quantitative detail of inter-word variability.	SPIDER: faster recognition (score = **-0.69**); ANTEATER: slower recognition (score = **0.10**).	As with prevalence, the English Crowdsourcing Project (Mandera et al., 2020).
*Valence*	This feature indicates the level of pleasantness evoked by the word. The score ranges from 1 to 9.	WASP: lower valence (score = **2.71**); PANDA: higher valence (score = **7.55**).	Warriner et al., 2013; although pleasantness of words is a subjective trait, rating dispersion was relatively low.
*Arousal*	This feature indicates the strength of the emotion induced by the word. The score ranges from 1 to 9.	SEAL: lower arousal (score = **2.50**); CROCODILE: higher arousal (score = **6.48**).	Warriner et al., 2013
*Dominance*	This feature indicates the level of perceived control towards the referent. The score ranges from 1 to 9.	BEAR: lower dominance (score = **3.59**); BULL: higher dominance (score = **6.89**).	Warriner et al., 2013
*Body-Object Interaction*	This feature (scored onto a scale from 1 to 7) quantifies the possibility offered by the referent of a word to be interacted with. It is a semantic quality that embodies the sensorimotor information associated with a certain word (Hargreaves et al., 2012).	PLATYPUS: lower body-object interaction (score = **3.04**); DOG: higher body-object interaction (score = **6.40**).	Pexman et al., 2019
**Non-semantic Features**			
*Graphemes Count*	The orthographic transcription of the word was scored. Spaces separating two terms (e.g., as in “GUINEA PIG” or “PASSION FRUIT”) were not counted.	OX: shorter word (**2** graphemes); CATERPILLAR: longer word (**11** graphemes).	N/A
*Syllables Count*	Although strongly correlated with the number of graphemes, this feature was included as there are examples of common words in which this correspondence is invalid.	IGUANA: **3** syllables (with 6 graphemes); SHRIMP: **1** syllable (with 6 graphemes).	N/A
*Consonant/Vowel Quantity Ratio*	This feature, meant to capture the ratio of consonant and vowel quantity, represents a basic phonological descriptor expected to be completely unrelated to the difficulty of word retrieval. The scoring was carried out on the UK phonetic transcription of the word.	BUFFALO (“bΛfələʊ”): 7 phonemes 3 of which are consonants = **0.43**.	Dufau et al., 2015
*Phonological Complexity*	Complexity of consonant clusters was scored based on the UK phonetic transcription of the word, following the model of consonant sonority and scoring proposed by Riley and Thompson (2015). As word length may influence this feature (i.e., the longer the word, the more consonants there may be), the additive complexity score of all clusters within a word was partialised by the number of syllables.	PHEASANT (“fεzənt”): 3 consonant clusters. 1) “f”, voiceless fricative, sonority of 5; 2) “z”, voiced fricative, sonority of 4; 3) “nt”: combination of a nasal occlusive, sonority of 3, and a voiceless stop, sonority of 7: combined sonority of 4. Global score = 13. Partialised score (2 syllables) = **6.5**.	Riley and Thompson, 2015
*Serial Recall Order*	An incremental score from 1 to *n* was assigned to each correct entry (from the first to the last) generated for each category. This variable reflects the serial order with which words are recalled via the semantic cue assigned and is expressed as an ordinal scale.	e.g., CAT (**1**), DOG (**2**), HORSE (**3**), SHEEP (**4**), DUCK (**5**), SWAN (**6**), LION (**7**), TIGER (**8**), GIRAFFE (**9**) …	N/A
*In-List Orthographic Levensthein Distance*	This feature is a metric of similarity between two orthographic strings (Yarkoni et al., 2008). Each word was compared to every other word generated by the participant to obtain word-to-word distances based on the minimum number of graphemes that would need to be replaced/removed/inserted. An average distance was then calculated for each word in relation to all other words.	PARROT (target word); HORNET (comparison word 1): distance = 4; PANTHER (comparison word 2): distance = 5; OCELOT (comparison word 3): distance = 4; average distance = 4.33. Underlined are the elements of difference that constitute the distances.	Scoring was carried out through the resources provided at https://www.dcode.fr/levenshtein-distance.
*Dictionary Orthographic Levensthein Distance*	This feature is a metric of the ‘orthographic neighbourhood’ of a word. Levensthein Distances were calculated to establish the number of terms in the entire English dictionary differing from the target word by one grapheme.	OTTER (target word); number of words that differ by one grapheme = 7: UTTER, OTTERS, HOTTER, POTTER, OUTER, OTHER, COTTER. Underlined are the elements of difference that constitute the distances.	As with the previous feature, scoring was carried out via the resources provided at https://www.dcode.fr/levenshtein-distance.

### Feature-to-Feature Correlations

**FIGURE 2:**
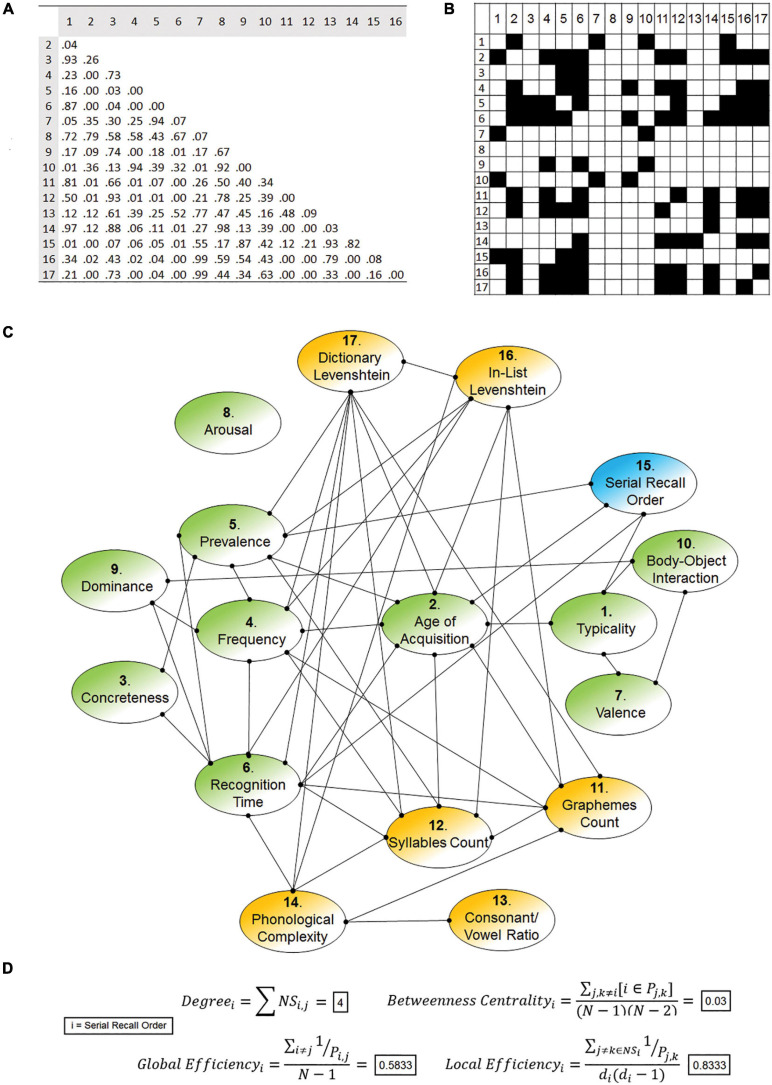
Example of matrices and graph calculated on a single participant (a 71 year-old woman). The feature-to-feature correlational matrix **(A)** and the binary adjacency matrix tagging significant correlations **(B)** are shown. Please note that, since based on correlations, adjacency matrices express bidirectional relationships. The graph **(C)** colour-codes and distinguishes the node of interest (blue) from the ten semantic features (green) and the other non-semantic features (yellow). Nodal metrics of “serial recall order” (inclusive of formulas) for this specific participant are reported in the lower part of the image **(D)**.

**FIGURE 3:**
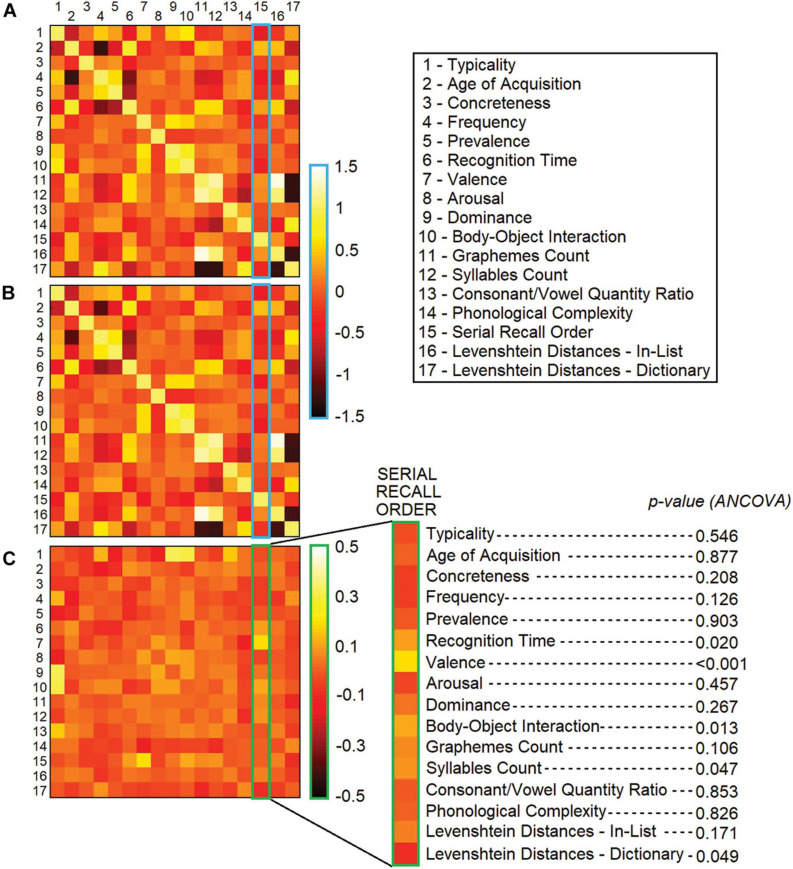
*z*-transformed coefficients of correlation calculated across the entire 17 × 17 matrix within the group of older adults **(A)** and younger adults **(B)**. Between-group difference scores (where scores among older and younger adults are the subtrahend and minuend, respectively) are shown below **(C)**, flanked by the outcome of statistical comparisons. Blue and green frames were added to highlight the coefficients of correlation relevant to this study.

Once scoring was completed for all items, the two categories (animals and fruits) were merged to maximise the size of individual data distributions. Coefficients of non-parametric correlation (*Spearman’s rho*) were thus calculated to compute all 136 patterns of feature-to-feature association (Figure 2), i.e., [(*n*×(*n*−1)]/2 = 136. In case of missing data (i.e., words with no available rating for a specific feature), correlational models were run with the remaining available values. The count and proportional implications of missing data were reviewed throughout the cohort. Each participant had between 19 and 43 observations per each of the 17 features for the calculation of individual correlational profiles, with medians ranging between 30 (for valence, arousal, and dominance) and 33.5 (for typicality) observations. Only 16 of the 136 feature-to-feature correlations were analysed to comply with the first methodological approach (i.e., the correlation between SRO and the other 16 features; see Figure 3 for details on the 16 correlational patterns of interest), while the remaining 120 feature-to-feature correlations were not considered any further. These additional correlations, in fact, are unrelated to memory, but simply describe associations among pairs of semantic and non-semantic features (e.g., between “graphemes count” and “body-object interaction”) that are of no direct interest to the study of SRO. To allow between-group inferential statistics, all coefficients were converted to *z*-scores, by applying a *Fisher’s rho-to-z transformation* (Zar, 2005, Eq. 19).

All 16 distributions of feature-to-feature *z*-converted correlation coefficients were tested for normality (*Shapiro–Wilk* test), presence of outliers [the method recommended by Hoaglin, Iglewicz, and Tukey based on a 2.28 × *IQR* cut-off (Hoaglin et al., 1986)] and between-group homogeneity of variance (*Levene*’s test). There were no missing data in these analyses.

### Graph-Theory Analysis of Correlations

Commonly used in neuroimaging to analyse the complexity of brain networks (Bullmore and Sporns, 2009), graph theory is a mathematical framework that studies systems of variables related to each other in various (direct and indirect) ways. A graph is usually represented in the form of a schematic illustration in which variables are arranged in the two-dimensional space and connected to one another with a series of lines (Figure 2C). Variables are indicated as “nodes” of the graph while the word “edge” refers to a link that connects any two nodes on the basis of some established relationship. A third important concept is that of “neighbouring sub-graph” of a node (“*NS*”, in the equations below), that is the set of nodes connected to it with an edge. Subject-specific graphs of 17 nodes were created and, to ensure that graphs included only significant node-to-node associations, the edge-forming rule was chosen based on the significance level of the correlation coefficients. To this end, two thresholds of significance were considered (*p* < 0.05 and *p* < 0.01). All edges were unweighted (i.e., having the same value) and undirected (i.e., expressing a significant, non-directional coefficient of correlation). Figures 2A–C illustrates an example of subject-specific graph, where edge-defining correlations were calculated in a dataset obtained from the administration of the CFT to a single individual.

Four metrics were calculated to characterise the node of interest (i.e., SRO): *degree*, *betweenness centrality*, *global efficiency*, and *local efficiency*. The arithmetical formula of each metric (Rubinov and Sporns, 2010) for a node “*i*” is as follows (i.e., consult Figure 2D for a practical application of these four formulas on an individual CFT graph):

$$Degree_{\text{i}}=\sum NS_{i,j}$$

The degree of a node is the sum of all edges linking it to other nodes (i.e., the number of significant correlations),

$$BetweennessCentrality_{\text{i}}=\frac{\sum_{j,k\neq i}\left[i\in P_{j,k}\right]}{\left(N-1\right)\left(N-2\right)}$$

while its betweenness centrality is a fractional measure of the number of times the node is part of the shortest path (measured in number of edges; “*P*” in the formula) that connects any two nodes of the graph (“*j*” and “*k*”). These two metrics were used as indices of direct centrality (degree) and global centrality, i.e., the central role played by nodes within the whole graph (betweenness centrality), respectively.

$$GlobalEfficiency_{\text{i}}=\frac{\sum_{i\neq j}1/P_{i,j}}{N-1}$$

Global efficiency of a node (an index of integration) is a proportion of the number of nodes of the graph and consists of the inverse of the average shortest path that links the node in question to the other nodes.

$$LocalEfficiency_{\text{i}}=\frac{\sum_{j\neq k\in NS_{i}}1/P_{j,k}}{d_{i}\left(d_{i}-1\right)}$$

Local efficiency of a node is instead a proportion of the node’s degree (“*d*”, in the above formula) and consists of the inverse of the average shortest path between each pair of nodes that are part of the neighbouring sub-graph of interest (minus the node of interest itself).

To assess the performance of the two edge-forming rule candidates (i.e., correlations significant at a *p* < 0.05 or 0.01), indices of *cost efficiency* were calculated (the *cost* of a node is equal to its degree divided by N–1). These were not calculated for a single node (as with the formulas above) but for the entire graph (i.e., via an average of all nodal measures).

CostEfficiency=GlobalEfficiency-Cost

A *p*-value < 0.05 was associated with a significantly more convenient cost efficiency (*t*_89_ = 23.201, *p* < 0.001; paired-sample *t*-test), and was thus retained as the edge-forming rule for this study. This choice resulted in a number of edges between 23 and 64 (out of 136) in the two cohorts (younger adults: *mean* = 43.71, *SD* = 7.84; older adults: *mean* = 46.58, *SD* = 8.69; there was no between-group difference). The calculation of these indices was carried out using the Brain Connectivity Toolbox^1^, implemented in MATLAB (R2014a, Mathworks Inc., United Kingdom).

### Statistical Inference

To address the first hypothesis, coefficients of correlation (*Spearman’s rho*) were run to test the association between standard and correlational CFT indices of interest and two measures selected from the neuropsychological battery: the Digit Cancellation Test (Della Sala et al., 1992) as a measure of processing speed and the Stroop Test–Time Interference (Venneri et al., 1992) as a measure of executive functioning. A conservative *p*-value < 0.01 was used as statistical threshold.

To address the second hypothesis, one-way analyses of covariance (ANCOVAs) were run to compare the correlational profiles of younger and older adults. Both *z*-transformed correlation coefficients and graph metrics were analysed. Each model was corrected for years of education as a proxy of cognitive reserve (Stern, 2009), Mini-Mental State Examination score (Folstein et al., 1975) as an index of overall cognitive functioning and raw CFT score to control for the variability in the number of entries at the basis of the correlation. These were all included as covariates. As above, a conservative *p*-value < 0.01 was used as statistical threshold in the analyses of *z*-transformed coefficients of correlation. Given the novelty and the exploratory nature of the graph-metrics approach, a more lenient *p*-value of 0.05 was instead used as threshold of significance in the analysis of graph theory metrics.

## Results

The application of study criteria resulted in the recruitment of 250 healthy controls resident in the United Kingdom Yorkshire and Humber region, including 45 younger adults aged 18–21 years old (who were all entered in this study) and 75 older adults aged ≥70 years old, 45 of whom were randomly selected for this investigation. The demographic and cognitive profile of the two groups is included in Table 1. All participants were monolingual English native speakers of White-British ethnicity who were born and had their educational training in the United Kingdom. They all took part in the data collection on a voluntary basis and received no compensation or academic credits in return.

### Cognitive Profiles

The classification of test performance carried out in the group of older adults using the framework by Guilmette and co-authors (Guilmette et al., 2020) revealed that the majority (∼85%) of test scores was “*within normal limits*,” with a further ∼10% of “*low average*,” ∼5% “*below low average*” and less than 1% “*exceptionally low*” scores. This was consistent with rates expected in healthy controls assessed with a multi-test battery (Axelrod and Wall, 2007; Binder et al., 2009). In addition, none of the participants met the criteria for a diagnosis of mild cognitive impairment. Table 1 illustrates the cognitive profiles of the two groups. Younger adults performed significantly better on tests of long-term episodic memory (Paired Associated Learning Test and the recall of the Rey-Osterrieth Complex Figure), visuo-constructive abilities (Visuoconstructive Apraxia Test and the copy of the Rey-Osterrieth Complex Figure) and attentive/inhibitory skills (Digit Cancellation Test and Stroop Test time interference), while older adults scored significantly better on tests measuring lexical/semantic processing and SM (Letter Fluency Test, Confrontational Naming Test, and Pyramids and Palm Trees Test). These group differences are in line with the trends commonly seen in association with normal ageing. Performance on the Stroop test (arguably the task in the battery with the highest cognitive demands) indicated time-interference latencies <46.5 s and <25 s in the group of older and younger adults, respectively, suggesting satisfactory levels of commitment during task performance. In addition, as performance on the Raven’s Coloured Progressive Matrices is often used as a proxy of general non-verbal IQ (Wongupparaj et al., 2015), an inspection of scores on this test indicated normal intelligence in all participants.

In total, 3311 words were generated by the entire cohort as part of the CFT, including 254 (7.7%) perseverations and 20 (0.6%) intrusions. No group differences on the CFT were found either when “animals” and “fruits” were analysed separately, or when they were combined. The analyses of cross-category consistency revealed a significant linear association across the whole cohort, with valid “animals” entries significantly predicting the number of valid “fruits” (*b* = 0.339). Trends in the same direction were found when analyses were run separately in each age group, with older adults showing a weaker association (*b* = 0.205) and younger adults showing a stronger association (*b* = 0.634). A visual representation of these linear associations and the results of a validation analysis carried out in an independent cohort are reported in the Supplementary Material.

### Feature-to-Feature Correlations

Fifteen out of 16 distributions of feature-to-feature correlational scores met the assumptions of normality. The only distribution in breach of the assumption was that of the *z*-converted correlation coefficient between SRO and age of acquisition. This was also the only distribution in which an outlier (an older adult) was detected. After removing the outlier, the assumption was met. In addition, between-group homogeneity of variance was confirmed for all but three correlational features: those between SRO and concreteness, prevalence and dictionary orthographic Levenshtein distance. In all three cases older adults had a wider distribution with a total of five extreme values located at a >1.5 × *IQR* distance from the upper/lower quartile. After removing these five data-points, the assumption was met.

The standard CFT score was significantly correlated with performance on the Digit Cancellation Test (*rho* = 0.279, *p* = 0.002). None of the SRO-based correlations was associated with performance on the Digit Cancellation Test or Stroop Test–Time Interference.

**FIGURE 4:**
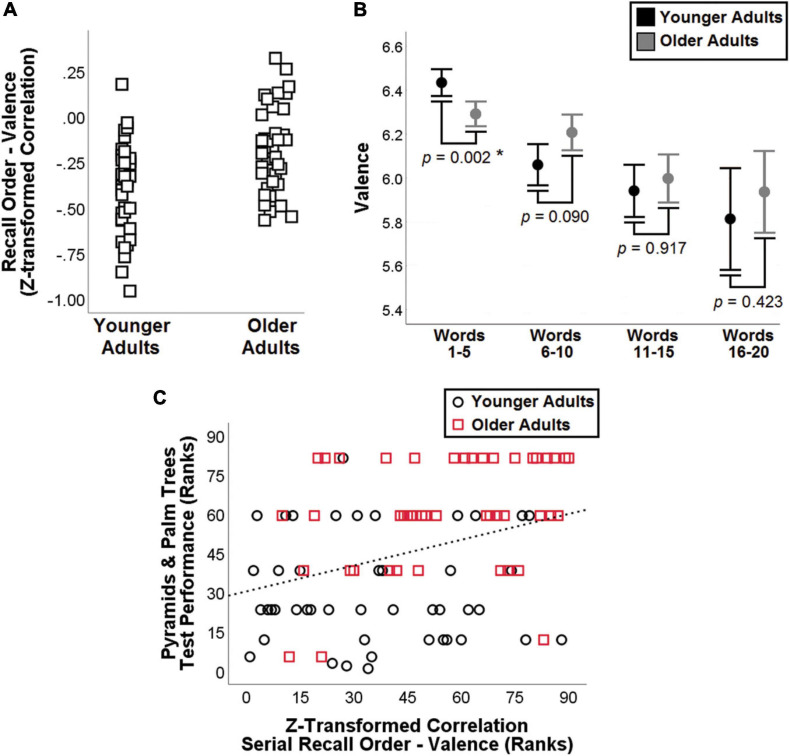
Outcome of feature-to-feature correlation analysis. Group distributions of the z-transformed coefficient of correlation between serial recall order and valence is shown on the left **(A)**, while *post hoc* analyses of five-word positions are shown on the right **(B)**. The association between ranked z-transformed correlation coefficients and performance on the Pyramids and Palm Trees Test is shown below **(C)**.

The direction of the association (i.e., the sign of the correlation coefficient) was the same in both groups for all 16 models. Only one standardised correlation coefficient out of the pool of 16 differed between the two groups, i.e., that between SRO and valence (*F*_1,85_ = 15.979, *p* = 0.00014, η^2^*_*p*_* = 0.158; Figure 3). This association was still significant even when the analysis was corrected for all other 15 *z*-transformed correlation coefficients, included as covariates (*F*_1,70_ = 14.255, *p* = 0.00033, η^2^*_*p*_* = 0.172). As words were recalled, the decrease in valence was steeper in younger adults. To characterise this pattern more in detail, words retrieved in positions 1–5, 6–10, 11–15, and 16–20 were grouped together for *post hoc* analysis. *ANOVA* models were thus designed to test the effect of age group on each positional set, controlling for years of education and Mini-Mental State Examination score (the raw CFT score was not included as a covariate in these models as it is a property of the entire 1-min performance and is unrelated to the words generated in each positional set). Only words in position 1–5 differed between the two age groups, with younger adults retrieving words of significantly higher valence (*p* < 0.001, η^2^*_*p*_* = 0.122; Figure 4). The words most commonly generated by the two groups in position 1–5 are reported in Table 3. When positional sets were analysed for each separate category, animals 1–5 showed a significant difference (*p* = 0.004, η^2^*_*p*_* = 0.094) while only a trend was observed for fruits 1–5.

**TABLE 3:** Words generated by each group in position 1–5.

Younger Adults				Older Adults			
Word	Count	Valence: Global Normative Score (Younger Adults Score)	Age Difference	Word	Count	Valence: Global Normative Score (Older Adults Score)	Age Difference
**Animals**							
DOG	39	7.00 (7.09)	–0.09	DOG	33	7.00 (6.89)	0.11
CAT	38	6.95 (6.50)	0.45	CAT	31	6.95 (7.40)	–0.45
LION	12	5.84 (6.10)	–0.26	COW	17	5.42 (5.40)	0.02
MOUSE	12	4.80 (4.75)	0.05	HORSE	12	6.05 (6.21)	–0.16
FISH	10	6.42 (6.43)	–0.01	MOUSE	12	4.80 (4.83)	–0.03
HAMSTER	10	5.88 (6.44)	–0.56	PIG	12	4.83 (4.78)	0.05
HORSE	10	6.05 (5.83)	0.22	SHEEP	12	5.32 (5.10)	0.22
RABBIT	10	7.21 (6.89)	0.32	LION	10	5.84 (5.56)	0.28
BEAR	8	5.33 (5.36)	–0.03	GOAT	8	5.30 (5.10)	0.20
ELEPHANT	8	6.17 (5.57)	–0.40	RABBIT	7	7.21 (7.50)	–0.29
TIGER	8	6.00 (6.64)	–0.64	ELEPHANT	6	6.17 (6.55)	–0.38
GIRAFFE	7	6.52 (6.00)	0.52	RAT	6	3.21 (2.69)	0.52
RAT	6	3.21 (3.45)	–0.24	TIGER	5	6.00 (5.36)	0.64
SHEEP	6	5.32 (5.56)	–0.24	COW	5	5.42 (5.44)	–0.02
PIG	5	4.83 (4.89)	–0.06				
**Fruits**							
APPLE	44	6.62 (7.25)	–0.63	APPLE	40	6.62 (6.47)	0.15
BANANA	34	6.71 (6.56)	0.15	ORANGE	34	6.81 (7.00)	–0.19
PEAR	33	6.70 (6.80)	–0.10	PEAR	29	6.70 (6.60)	0.10
ORANGE	29	6.81 (6.43)	0.38	BANANA	25	6.71 (7.20)	–0.49
GRAPE	14	6.70 (6.27)	0.43	GRAPE	9	6.70 (7.22)	–0.52
KIWI	8	6.11 (6.50)	–0.39	LEMON	9	6.37 (6.20)	0.17
PINEAPPLE	8	6.90 (6.33)	0.57	GRAPEFRUIT	7	5.77 (6.00)	–0.23
STRAWBERRY	8	7.25 (6.91)	0.34	PEACH	6	6.83 (7.20)	–0.37
MANGO	6	6.57 (7.75)	–1.18	MELON	5	6.32 (6.23)	0.09
PEACH	5	6.83 (6.38)	0.45	PLUM	5	6.15 (6.20)	–0.05
TOMATO	5	5.80 (5.00)	0.80				

*Counts are to be intended out of 45, that is the total number of participants per group, e.g., 39 younger adults and 33 older adults out of 45 generated “*dog*” among the first five recall positions. Frequencies of 4 and less are not shown.*

### Graph-Theory Analysis

**FIGURE 5:**
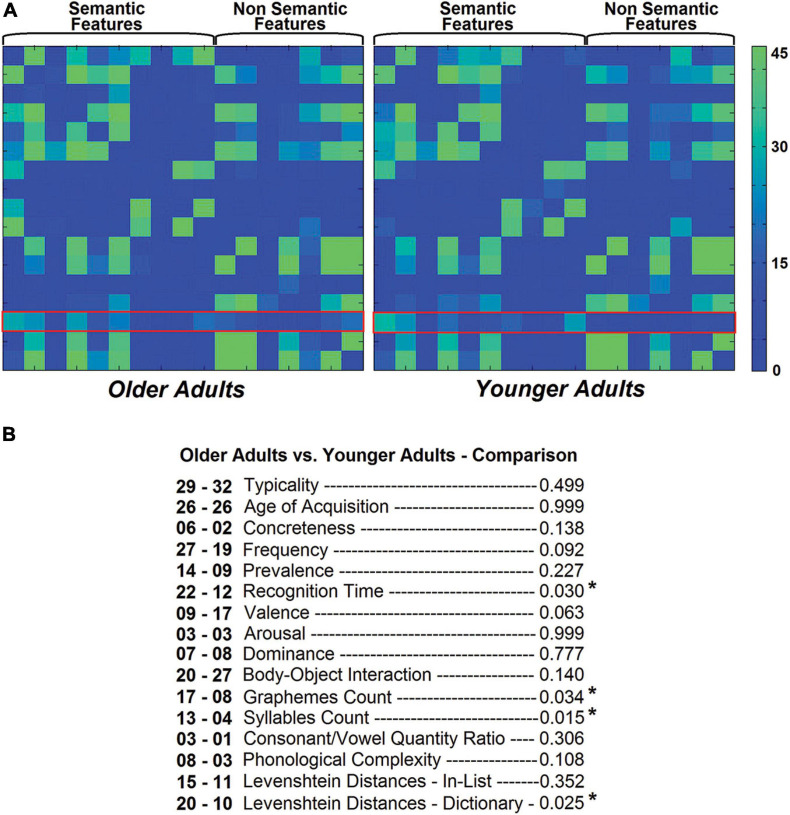
Edge frequency in the two groups. A red frame was added to highlight the edges relevant to this study **(A)**. A count of all these edges within each group is included below together with the outcome of the *chi-square* tests comparing edges between the two group frequencies, older and younger adults, respectively **(B)**. Four pathways showed significant between-group differences. These same pathways approached or showed a trend toward significance when z-transformed correlation coefficients were analysed, as illustrated in Figure 2. Similarly, the edge toward valence approached significance in these analyses.

Nodal properties of SRO were extracted from each subject-specific graph for the purpose of group-level analyses. Edge frequency in the two groups is illustrated in Figure 5. The SRO node counted a total of 431 edges across the whole cohort (older adults: 239, younger adults: 192), 318 of which (∼ 74%) were toward a semantic node. The five nodes most often correlated (and thus expressing an edge) with SRO were typicality (61 times out of 90), age of acquisition (52 times), body-object interaction (47 times), frequency (46 times) and recognition time (34 times). The five least frequently correlated nodes were instead consonant/vowel quantity ratio (4 times), arousal (6 times), concreteness (8 times), phonological complexity (11 times), and dominance (15 times). A series of *chi-square* tests were run to compare edge frequency between the two groups. Older adults had more edges between SRO and recognition time (φ = 0.229), graphemes count (φ = 0.223), syllables count (φ = 0.255) and the orthographic Levenshtein distance between words and dictionary entries (φ = 0.236); all *p*-values < 0.05.

Statistical differences for the node of interest between the two groups were found in two of the four metrics: degree and betweenness centrality (Table 4A). SRO was characterised by significantly lower betweenness centrality (*F*_1,85_ = 4.002, *p* = 0.049, η^2^*_*p*_* = 0.045) and by higher degree (*F*_1,85_ = 4.323, *p* = 0.041, η^2^*_*p*_* = 0.048) in the group of older adults. Younger adults had an average of 4.24 edges connecting SRO to other nodes, while older adults had an average of 5.29. The count of the edges from SRO toward semantic nodes, however, was similar between groups (older adults: *mean* = 3.64, *SD* = 1.57; younger adults: *mean* = 3.44, *SD* = 2.00). Metric-to-metric correlation coefficients (*Pearson’s r*) are reported in Table 4B.

**TABLE 4:** Metrics calculated in association with the “serial recall order” node (A) and metric-to-metric associations.

Variable	Younger Adults	Older Adults	*P*
***(A) SRO Nodal Metrics***			
Degree	4.24 (2.24)	5.29 (3.17)	0.041*
Betweenness Centrality	0.09 (0.12)	0.05 (0.06)	0.049*
Global Efficiency	0.54 (0.15)	0.56 (0.19)	0.353
Local Efficiency	0.67 (0.32)	0.67 (0.36)	0.981
***(B) Correlations Among SRO Metrics***			
	Degree	Local Efficiency	Global Efficiency
Local Efficiency	0.369***		
Global Efficiency	0.870***	0.397***	
Betweenness Centrality	0.397***	−0.167	0.420***

*(A) Means and standard deviations are indicated. Inferential models are described in text.*

(B) Pearson’s coefficients of correlation are reported.

*: *p* < 0.05; ***: *p* < 0.001.

### Link Between Significant Metrics and Cognitive/Demographic Variables

To explore the association between the 20 metrics investigated in this study (16 feature-to-feature *z*-transformed correlations and four nodal graph-theory metrics) and performance on standard cognitive tests (those included in the “Neuropsychological Assessment” sections of Table 1, other than Digit Cancellation Test and Stroop Test–Time Interference), coefficients of correlation were calculated at *post hoc* within the entire group of 90 adults using a Bonferroni-corrected *p* < 0.0025 (0.05/20) and controlling each model for the same covariates as in the main analyses (*Spearman’s* coefficient of partial non-parametric correlation). One sole correlation retained statistical significance: the *z*-transformed coefficient of correlation between SRO and valence was significantly correlated with performance on the Pyramids and Palm Trees test (*rho*_85_ = 0.333, *p* = 0.002). Associations significant at an uncorrected, more lenient *p* < 0.05 are illustrated in the Supplementary Material.

We also tested the association between the 20 outcome metrics and the number of intrusions and perseverations made by participants during CFT. No model was significant at a Bonferroni-corrected *p* < 0.0025. Associations significant at an uncorrected, more lenient *p* < 0.05 are illustrated in the Supplementary Material.

Finally, we tested the association between the 20 outcome metrics and three major demographic variables: education, Mini-Mental State Examination score and sex, using the same threshold of significance. Education was significantly correlated with the *z*-transformed coefficient of correlation between SRO and Graphemes count (*r*_90_ = −0.344, *p* = 0.001), while general cognitive functioning measured via the Mini-Mental Examination Score was significantly correlated with two nodal indices of graph theory: SRO degree (*rho*_90_ = 0.323, *p* = 0.002) and SRO global efficiency (*rho*_90_ = 0.321 *p* = 0.002). As sex had a binary distribution, differences between males and females were tested with *t-*tests. No between-group differences, however, emerged as significant. Associations significant at an uncorrected, more lenient *p* < 0.05 are illustrated in the Supplementary Material.

## Discussion

The study of SM is of particular interest to cognitive neuroscientists. There is, however, a methodological need for fine-grained measures of SM that are not excessively influenced by other functions. The CFT is often chosen by clinicians and researchers as preferred test of SM because, compared to other instruments (e.g., Boston Naming Test, Pyramids and Palm Trees/Camel and Cactus Test, the “Similarities” subtest of the Wechsler Adult Intelligence Scale, or tests based on recognition of famous people), it is a measure of free recall (Gruenewald and Lockhead, 1980) and does not require any adaptation for cross-cultural or cross-linguistic use. Differently from cued recall and recognition, free recall is a self-initiated form of retrieval more aligned with real-life scenarios (Craik, 1983), and this confers a degree of ecological validity to this mode of testing. The CFT is also methodologically convenient, since it is simple and quick to administer and does not require a complex set-up. Moreover, it can be transposed into any language without requiring complex translations or validation studies. Facilitated by these aspects, it has proven to be a particularly versatile test, since a considerable number of innovative scoring procedures have been put forward, in an attempt to improve and optimise test measures that can be of assistance in clinical practice. In line with this goal, in this study we have devised a scoring method that combines the serial order of CFT word retrieval with the semantic “difficulty” of each word, quantified as a function of 16 separate semantic and non-semantic features. To put the validity of this profile of correlational variables to the test, we formulated a first hypothesis based on which correlational indices linking SRO to semantic features would be less statistically associated with performance on tests of speed of processing and executive functioning (functions that are known to support CFT performance) than the standard CFT score. We then formulated a second hypothesis addressing the effect normal ageing has on SM, with the expectation of a pattern of results aligned with older adults showing a more robust profile. To do so, we analysed the differences between younger and older adults, modelling *z*-transformed correlation coefficients in a direct way and indirectly, via the calculation of graph-theory metrics.

Although coefficients were similar between the two groups, the SRO-valence correlation indicated a robust difference (significant at a *p* < 0.001). *Post hoc* analyses showed that in the initial portion of the test (i.e., the first five words), older adults generated words of lower valence (i.e., typically perceived as less pleasant) than those generated by younger adults. While both age groups showed an overall decrement in valence as more words were generated, this decrease was steeper in the group of younger adults, as indicated by a significantly stronger coefficient of negative correlation. Experimental evidence indicates that there is a close relationship between SM and valence attribution (Bertoux et al., 2020). Other than showing consolidated semantic-memory skills (Nyberg et al., 1996, 2003; Park et al., 2002; Verhaeghen, 2003; Rönnlund et al., 2005; Small et al., 2011), older adults also show an “age-related positivity” effect, whereby stimuli of positive value have a processing advantage over stimuli of negative value (Reed and Carstensen, 2012). The combination of better SM and better processing of positive items indicates that older adults may be naturally prone to relying on valence during CFT performance. A similar trait does not characterise performance of younger adults, who show instead high level of valence only at the start of their performance (i.e., positions 1–5), when words are recalled with a high degree of automaticity and with limited need of semantic-control resources (Hurks et al., 2004) or strategies. We then tested whether age might play a role in the perceived valence of words. Evidence indicates that age is a significant, yet modest-at-best predictor of attributed valence, with η^2^*_*p*_* effect sizes ranging from 0.001 (Söderholm et al., 2013) to 0.03 (Grühn and Smith, 2008), to 0.06 (Gilet et al., 2012), to an inferable *Cohen’s d* of 0.036 (Warriner et al., 2013). Our finding, however, cannot be ascribed to age differences in assigned valence because we relied on age-independent ratings, i.e., the same ratings were used for both groups (Warriner et al., 2013). We propose, therefore, that age differences exist in the degree to which automatic semantic retrieval is susceptible to pleasantness-related effects. There is experimental evidence that retrieval from memory is influenced by valence. The findings of an experiment carried out with younger adults showed that immediate recall of pleasant words is higher than immediate recall of neutral words (Monnier and Syssau, 2008). The representation of words with a positive or negative valence is semantically richer than that of neutral words, and pleasant words in particular also embed a “*life-enhancing*” connotation, enabling “*stronger semantic relatedness*” (Majerus and D’Argembeau, 2011, p. 182). This signifies that automatic semantic processing elicited by CFT in younger adults would tend to rely more on such “hedonistic” aspect. Although a precise explanation of the neural mechanisms that underpin this difference is beyond the scope of this study, research has highlighted that, differently from controlled elaboration of emotional content, automatic emotional processing of word stimuli involves the left hemisphere more than the right hemisphere (Abbassi et al., 2011). Functional asymmetries are typical of neurological processing and ageing is known to be associated with processes of dedifferentiation (Koen and Rugg, 2019), asymmetry reduction and recruitment of additional regions in support of task performance (Berlingeri et al., 2013). If lateralised specialisation during automatic verbal emotional processing is attenuated by age, this could play a pivotal role in accounting for the sharp difference in valence observed between the two groups in the first five-word interval. Nonetheless, older adults perform at the same level as younger adults without exploiting any valence-related boost at the start of the task. This may indicate optimised retrieval from SM that does not “impetuously” rely on a prominent feature that is limited to a short-lived effect. In support of the interpretation that more neutral and “stable” valence is indicative of better function, we found a positive correlation (the less steep the decline, the better the performance) between the *z*-transformed valence coefficient and performance on the Pyramids and Palm Trees Test, a non-verbal measure of SM unaffected by processing speed and with limited executive demands.

We acknowledge, however, that other, non-neurological factors might be at play. A close inspection of words retrieved in position 1–5 (Table 3) indicates that older adults retrieved more farm animals (i.e., *cow*, *horse*, *pig*, *sheep*, and *goat* were recalled 61 times by older adults and 34 times by younger adults) and fewer fruits typically considered “exotic” in the United Kingdom (i.e., *banana*, *kiwi*, *pineapple*, *mango*, *coconut*, and *papaya* were recalled, in total, 34 times by older adults and 58 times by younger adults). It is known that early socio-contextual exposures influence cognitive functioning in later life (Meyer et al., 2020). On these grounds, people in their 70s and 80s encoded semantic knowledge linked to animals and fruits at a time when society was not exposed to current modernisations [e.g., when animals mainly had a utilitarian function (Fogle, 1999) and when imported fruits were not as popular as endemic fruits]. As a consequence, we should not exclude that cross-sectional differences between younger and older adults might be due to multiple concurrent factors related to neurological processing as well as sociocultural differences. However, when global and age-specific ratings for word valence (Warriner et al., 2013) were compared (this was done for words in positions 1–5, where a significant group difference had emerged), no major deviation was found (Table 3), suggesting that, as far as these words are concerned, age does not seem to be associated with differences in valence attribution.

We also analysed the pattern of differences associated with SRO in a more exploratory way, following the principles of graph theory. This framework has already been used to analyse performance on the CFT, but only with nodes representing words and edges representing word-to-word, not feature-to-feature associations (Lerner et al., 2009; Goñi et al., 2011; Bertola et al., 2014). Operationalising CFT performance as a network of semantic and non-semantic features, SRO was characterised by higher degree and lower betweenness centrality at a liberal *p*-value < 0.05. Nodal degree, a simple metric of direct centrality, was higher in older adults, albeit not exclusively limited to edges toward semantic nodes. The number of edges between SRO and semantic features did not differ between the two groups and older adults had more often an edge between SRO and both semantic (recognition time) and non-semantic (graphemes count, syllables count and dictionary orthographic Levenshtein distance) nodes. Although these three latter features are devoid of semantic information (i.e., the number of letters and syllables and the number of existing words differing by one grapheme do not convey any semantic content) they do nonetheless show important connections with SM processing. Shorter words, for instance, tend to be acquired earlier in life (Łuniewska et al., 2019) and it is also known that words may activate the semantic information linked to their orthographic neighbourhood (Forster and Hector, 2002). Our findings thus suggest that semantic retrieval in older adults relies on additional lexical properties that are not semantic *per se*, but are of support in facilitating or expanding processing linked to SM retrieval. Conversely, although SRO betweenness centrality was positively correlated with SRO degree (Table 4B), it was lower among older adults. Although calculated in relation to each individual node, this metric captures a form of nodal centrality associated with the whole graph, quantifying the proportion of times the node of interest is part of the shortest path connecting any two nodes. Lower centrality in older adults indicates that, in this group, SRO played the role of mediator node a fewer number of times. Vice versa the role of SRO within the graph of younger adults tended to control and channel the statistical link among features significantly more often.

In summary, the use of correlational measures representing the association between SRO and semantic processing showed that older adults retrieve words tagging semantic content in a way that is emotionally more neutral and of increasing lexical and semantic richness and difficulty. This was not observed homogeneously for all aspects of semantic processing, but emerged only for certain features. The two approaches to data analysis were based on distinct profiles of association: *z*-transformed correlation coefficients were analysed as continuous outcome variables, while the associative links at the basis of the graphs were binarised after the application of a cut-off. This is probably the main reason why the features distinguishing the two groups differed between the two approaches. A trend of similarity, however, was observed across methodologies (see legend in Figure 5), ruling out sharp differences between the two methods and helping define in more detail the angle from which each pattern can provide independent information.

The goal of this study was to propose a novel approach to the analysis of the CFT. While a significant correlation was found between standard CFT performance and performance on the Digit Cancellation Test (indicating a link with speed of processing), none of the significant findings showed an association with performance on tests of executive functioning (e.g., Stroop Interference test) or processing speed (e.g., Digit Cancellation test), supporting the idea that the correlational operationalisation of target variables is less influenced by supporting/intervenient factors than standard CFT scoring. The outcome emerging from the direct modelling of correlational metrics was significantly associated with performance on a test of SM that is known to be minimally influenced by processing speed and executive functioning (the Pyramids and Palm Trees Test). These results provide further confirmatory evidence and suggest that, of the various semantic descriptors, valence appears to be that most susceptible to the effects of ageing.

A series of potential limitations is recognised. First, the number and variety of semantic and non-semantic features was the result of an arbitrary choice based on linguistic diversity and availability of reference ratings. Second, ratings were derived from diverse populations of native English speakers and were not exclusively based on British participants. Although variability undoubtedly exists across countries and across regional areas (e.g., the concept of “animal” in rural, coastal or urban areas) in the lexicon of the two categories explored in this study, we argue that this would not result in group-level differences in trends of correlation found in association with SRO. This is, however, a methodological aspect of further improvement. Third, although we combined animal and fruit entries to maximise the number of observations at the basis of the correlation coefficients, categories normally used as part of this test may show different levels of variability in their semantic features (Stokholm et al., 2013). The significant difference found in relation to positions 1–5 for valence was replicated for the “animals” category while a trend only emerged for the “fruits” category. We posit that this is linked to a larger variability in valence for the “animals” category (i.e., ranging from WASP: 2.71 to PANDA: 7.55, variance = 0.94) than for the “fruits” category (i.e., ranging from HAW: 4.35 to RASPBERRY: 7.30, variance = 0.18). “Animals” is among the most common categories used as part of the CFT, i.e., it is included in the “Addenbrooke’s Cognitive Examination Revised” and in the “Consortium to Establish a Registry for Alzheimer’s Disease” neuropsychological batteries. The findings of this study indicate that it is a category that offers a sufficiently sized variability to enable age differences in SM processing to emerge. Fourth, when the performance was subdivided into 5-word segments, a between-group difference was found only for the first segment. While this contributes to describing age-related trends, it is fair to note that this finding does not exploit the full lexical repertoire of the cohort, as it is based on the analysis of 900 words only (5 words × 90 participants × 2 categories), equal to only 29.4% of the total number of valid entries. Fifth, the sample was limited to 90 adults, a number that is insufficient to detect effects of small size. Sixth, although we had defined a stringent set of exclusions criteria to minimise the chances of recruiting ineligible participants, there are further neurological and psychological aspects uninvestigated in this study that may have contributed to account for part of the variability in the outcome measures. These include, for instance, genetic mechanisms (Savage et al., 2018), situational physiological variables (e.g., state anxiety/stress due to testing, mild partial sleep deprivation) and motivational factors. As far as motivation is concerned, however, although we did not administer any instrument explicitly designed to measure this process, a close inspection of individual performances on the Stroop Test (a task characterised by high cognitive demands) suggests sufficient levels of dedication put in this task by each participants. Finally, it is also worth noting that diagnoses were made based on the classification of uncorrected neuropsychological scores. Arguably, the introduction of corrected scores derived from normative data would improve diagnostic confidence and minimise the impact played by intervenient variables such as cognitive reserve.

Although this pattern of findings is preliminary at best, it warrants further attention to be paid to this theoretical framework. The additional findings obtained with the application of graph theory were significant at a more lenient threshold (*p* < 0.05) and are of exploratory relevance, given the novelty of the approach to feature-to-feature analyses. More work is needed to put additional aspects of this methodology to the test. This includes the study of test–retest reliability, its neuroimaging/neurophysiological correlates to verify construct validity, and the study of the influence additional demographic variables of neurological relevance may have, e.g., the mechanisms of cognitive reserve and plasticity. We anticipate that methods based on artificial intelligence (e.g., machine learning) could be an excellent route to process the large amount of correlational measures emerging from this procedures for a better characterisation of features that are of clinical relevance. Along the same lines, further methodological choices can be introduced to enrich the description of the link between SRO and semantic/non-semantic features, for instance the definition and assessment of Markov-Chain models to characterise in more detail the sequence of words generated during CFT. Further methodological steps could exploit the opportunity offered by statistics to isolate sources of variability by regressing out covariates of no interest or by applying latent-variable modelling to identify variables that cannot be directly measured.

This study investigated CFT performance in a group of adults with no neurological conditions. As a consequence, the extent to which this approach could be of help in clinical populations is still undetermined. Since, however, the methodology includes multiple outcome variables that are somewhat complementary to one another, these could be sensitive descriptors that could help detect very subtle neurological changes in SM or linguistic functioning (e.g., those that may occur during the pre-clinical phases of neurodegenerative conditions such as Alzheimer’s disease or frontotemporal lobar degeneration). Studies carried out in clinical populations are warranted to estimate the usefulness of this method in a clinical setting, as well as to define the possible use of computational algorithms to facilitate clinical use and adoption of this more innovative scoring approach.

In conclusion, these findings suggest that the application of our scoring methodology generates correlational measures that can be useful at describing SM according to multiple thematic and graph theory-informed metrics. Proof-of-concept analyses to test this scoring approach reveal that consolidation of SM typically occurring in normal ageing is detectable and characterisable with this approach. Of the 20 metrics analysed in this study, three yielded a significant difference suggesting an effect that is not general but specific to certain properties of SM. Similarly, it is expected that the same methodology might be effective at characterising decline of SM as seen in behavioural and neurodegenerative conditions.

## Data Availability Statement

The raw data supporting the conclusions of this article will be made available by the authors, without undue reservation.

## Ethics Statement

The studies involving human participants were reviewed and approved by the Regional Ethics Committee of Yorkshire and Humber, reference number 05/Q1104/129. The participants provided their written informed consent to participate in this study.

## Author Contributions

MDM conceived and designed the study, contributed to the literature search, data analysis, data interpretation, writing of the report, data curation, and contributed to the tables and the figures. DJB contributed to data interpretation. AV contributed to data collection and data interpretation. All authors contributed to the article and approved the submitted version.

## Conflict of Interest

The authors declare that the research was conducted in the absence of any commercial or financial relationships that could be construed as a potential conflict of interest.

## Publisher’s Note

All claims expressed in this article are solely those of the authors and do not necessarily represent those of their affiliated organizations, or those of the publisher, the editors and the reviewers. Any product that may be evaluated in this article, or claim that may be made by its manufacturer, is not guaranteed or endorsed by the publisher.

## Footnotes

1
https://sites.google.com/site/bctnet/Home/functions

## Abbreviations

CFT: category fluency test
SRO: serial recall order.
