# Supplementary material for: Serial Recall Order and Semantic Features of Category Fluency Words to Study Semantic Memory in Normal Ageing
Source: Front Aging Neurosci. 2021 Aug 3;13:678588. doi: 10.3389/fnagi.2021.678588 (PMC8370562; doi:10.3389/fnagi.2021.678588)
Supplement: Supplementary file 1 [file Data_Sheet_1.docx]

Serial Recall Order and Semantic Features of Category Fluency Words to Study Semantic Memory in Normal Ageing

Matteo De Marco^1,2^, Daniel J. Blackburn^1^, Annalena Venneri^1,2^

^1^ Department of Neuroscience, University of Sheffield, UK

^2^ Department of Life Sciences, Brunel University London, UK*****

**^*^Correspondence:**Matteo De Marco
[m.demarco@sheffield.ac.uk](mailto:m.demarco@sheffield.ac.uk)

Supplementary Material

# Category Fluency Test - Scoring Rules

The Category Fluency Test (CFT) is a free-recall task in which words belonging to certain categories have to be generated within a given time interval. The participant is assigned a category and, eventually, the list of words is scored. In this study the categories of reference were ‘animals’ and ‘fruits’. Two types of errors can be typically made during this task: intrusions and perseverations. We hereby provide the principles adopted to transform each word into its standardised entry and the rules defined to classify a word as an intrusion or perseveration.

- 1. **Standardised Entries**

To maintain a standard scoring procedure for all participants, a *standardised entry* was defined for each fruit and animal. This was based on the following procedure

- Each entry was transposed into its singular number;
- Two-word entries were coded as a one-word entry. Examples are POLAR BEAR, (standardised entry: BEAR) or REDCURRANT (standardised entry: CURRANT). By doing this, our intention was to avoid scoring of clusters like “BEAR, BROWN BEAR, POLAR BEAR, BLACK BEAR” or “CURRANT, WHITECURRANT, REDCURRANT, BLACKCURRANT” which are usually generated with very little semantic effort, and to avoid unnecessary missing data (since not all the databases we used as normative data included all two-word entries). In a number of cases the standardised entry retained its uniqueness, e.g., KOALA BEAR (standardised entry: KOALA), PANDA BEAR (standardized entry: PANDA), GRIZZLY BEAR (standardised entry: GRIZZLY) or CANTALOUPE MELON (standardised entry: CANTALOUPE). In a small proportion of cases, two-word standardised entries had to be defined, i.e., PASSION FRUIT, STAR FRUIT, GUINEA PIG and SEA LION.
- In three particular cases we encountered the same animal was expressed with two interchangeable terms: HIPPO-HIPPOPOTAMUS, RHINO-RHINOCEROUS and BUDGIE-BUDGERIGAR. The short form was selected as standardised entry in all these three cases since it was the more common.
  1. **Definition of Intrusion**

There are various ways in which the CFT can be approached. Professional zoologists and botanists may for instance approach the task with a certain level of technicality, while gardeners and bird watchers may focus on specific sub-categories prompted by their personal experience. Our intention was to devise a scoring procedure aligned with the idea of ‘animal’ and ‘fruit’ that could reflect that of the majority of the population. FRUIT, for instance, tends to refer to a “commercial” idea of fruit (or that of a cook), i.e., ‘what you would find in the fruit aisle of the supermarket’. CUCUMBER, for instance, refers to the fruit of the cucumber plant, but is not considered as a fruit in the traditional sense. The same applies to PEPPER and AUBERGINE. TOMATO was the only word often associated with vegetables that we accepted as a correct entry, given the large proportion of people in the UK who consider it to be a fruit. In this respect, the only nine words that were marked as intrusions in our cohort of 90 individuals were OLIVE, AUBERGINE, CUCUMBER, PEPPER, SQUASH, HOP, GROCER, HUMAN BEING and PTERODACTYL. It was decided not to accept as valid entries words indicating extinct or imaginary animals (e.g., TYRANNOSAURUS or UNICORN). Although biologically correct, HUMAN/HUMAN BEING was also marked as an intrusion.

- 1. **Definition of Perseveration**

While we recorded a fairly limited number of intrusions (additional intrusions were generated as part of the CITIES category), the number of entries classified as perseverations was considerably higher. In a certain proportion of cases the participant generated the exact same entry more than once, or an animal/fruit corresponding to the same standardised entry of a word already given. Other times, the participant generated an entry that, for scoring purposes, could not coexist with an entry already given. In this latter case these rules were applied to define a perseveration:

- Super-ordinate or subordinate entries of an entry already given: if the participant gave SHARK and then, later in the task, FISH (a superordinate term). Or, alternatively, if the participants gave MONKEY and then LEMUR (a subordinate term). An example valid for the fruit category is APPLE and GRANNY SMITH. In all these cases the first word is accepted and the subsequent one(s) is/are not and are flagged as perseverations.
- An entry that refers to an animal/fruit that has already been given, but in a different context, e.g., GRAPE and RAISIN (normal and dehydrated), SHEEP and LAMB (adult and young), BULL and COW (male and female). As above, in these cases the first word is accepted and the subsequent one(s) is/are not and are flagged as perseverations. A further potential example worth of consideration (but not observed in out cohort) is that of animals at a larval and adult stage, e.g., TADPOLE and FROG or CATERPILLAR and BUTTERFLY.

It is worth remarking that these procedures are only meant to set scoring standards and that many of the rules described above may be considered arbitrary. On this note, we expect that a reasonably different choice of rules (e.g., super/subordinate words being allowed) would have very little or no effect on the global correlation scores and on the adjacency matrices calculated for graph theory analysis. In the specific case of this study, the choice of these rules was informed in a substantial way by the list of entries for which normative data were available. For instance, of all normative studies listed in **Table 2**, the SUBTLEX-UK initiative (1) is by far that with the largest database of scores (*n* > 160K), yet, it does not include many common two-word entries such as PANDA BEAR. For this reason, we reached a compromise and outlined a sequence of scoring rules that could be reasonable and that could, at the same time, maximise the number of available scores for the calculation of the coefficients of correlation.

1. **Cross-category consistency**

The linear association between the number of ‘animals’ and ‘fruits’ entries was analysed with regression models. Scatterplots inclusive of regression lines for the entire cohort and for each age group are shown in **Figure S1**. Since the regression line calculated in the group of older adults was not as steep as in the group of younger adults, an additional validation analysis was run using CFT data published by the Alzheimer’s Disease Neuroimaging Initiative (ADNI)-1 (<http://adni.loni.usc.edu/>). Two-hundred-and-twenty older adults (aged 70 or above) of comparable Mini-Mental-State Examination scores as our group of older adults (*mean* = 29.12, *SD* = 1.00) completed a CFT based on two categories analogous to those included in this study: ‘animals’ and ‘vegetables’. The slope of the regression line in this supplementary analysis indicates a strong linear association between the two categories (*b* = 0.587), providing strong support for the validity of cross-category procedures in this age group.

1. **Link between outcome metrics and cognitive performance**

In addition to the findings described in **Section 3.4** of the main manuscript illustrating *post hoc* correlations between outcome variables and the tests used to characterise cognitive profiles (i.e., those listed in **Table 1**), additional findings were significant at a *p* < 0.05 uncorrected for multiple comparisons. These are as follows (i.e., the only model significant at a Bonferroni-corrected *p* < 0.0025 reported in the main manuscript is indicated with *******):

- 1. **Feature-to-feature correlational outcomes**
- ‘SRO-Typicality’ - Rey Osterrieth Complex Figure Test-Copy (*rho_85_* = -0.225, *p* = 0.036);
- ‘SRO-Concreteness’ - Pyramids and Palm Trees Test (*rho_85_* = -0.276, *p* = 0.010);
- ‘SRO-Frequency’ - Letter Fluency Test (*rho_85_* = -0.275, *p* = 0.010);
- ‘SRO-Frequency’ - Token Test (*rho_85_* = -0.322, *p* = 0.002);
- ‘SRO-Prevalence’ - Digit Cancellation Test (*rho_85_* = -0.247, *p* = 0.021);
- ‘SRO-Prevalence’ – WAIS-Similarities Test (*rho_85_* = -0.232, *p* = 0.031);
- ‘SRO-Recognition Time’ - Confrontation Naming Test (*rho_85_* = 0.229, *p* = 0.033);
- ‘SRO-Valence’ - Confrontation Naming Test (*rho_85_* = 0.304, *p* = 0.004);
- ‘SRO-Valence’ - Pyramids and Palm Trees Test (*rho_85_* = 0.333, *p* = 0.002) *******;
- ‘SRO-Valence’ - Rey Osterrieth Complex Figure Test-Copy (*rho_85_* = -0.241, *p* = 0.025);
- ‘SRO-Valence’ – Stroop Test-Time Interference (*rho_85_* = 0.303, *p* = 0.004);
- ‘SRO-Dominance’ - Confrontation Naming Test (*rho_85_* = 0.216, *p* = 0.044);
- ‘SRO-Body Object Interaction’ - Pyramids and Palm Trees Test (*rho_85_* = 0.216, *p* = 0.044);
- ‘SRO-Graphemes Count’ - Token Test (*rho_85_* = 0.238, *p* = 0.027);
- ‘SRO-Syllables Count’ - Token Test (*rho_85_* = 0.314, *p* = 0.003);
- ‘SRO-In-List Levenshtein’ - Token Test (*rho_85_* = 0.247, *p* = 0.021);
- ‘SRO-Dictionary Levenshtein’ – Stroop Test-Time Interference (*rho_85_* = -0.245, *p* = 0.022);
- ‘SRO-Dictionary Levenshtein’ - Token Test (*rho_85_* = -0.255, *p* = 0.017);
- ‘SRO-Typicality’ - Perseverations on the Category Fluency Test (*rho_85_* = -0.225, *p* = 0.036);
- ‘SRO-Frequency’ - Perseverations on the Category Fluency Test (*rho_85_* = -0.230, *p* = 0.032);
- ‘SRO-Recognition Time’ - Perseverations on the Category Fluency Test (*rho_85_* = 0.273, *p* = 0.011);
  1. **Graph theory-informed outcomes**
- ‘SRO-Local Efficiency’ – WAIS-Similarities Test (*rho_85_* = 0.271, *p* = 0.011);

1. **Link between outcome metrics and demographic variables**

In addition to the findings described in **Section 3.4** of the main manuscript illustrating *post hoc* associations between outcome variables and the three main demographic variables (other than age) listed in **Table 1**, additional findings were significant at a *p* < 0.05 uncorrected for multiple comparisons. These are as follows (i.e., the models significant at a Bonferroni-corrected *p* < 0.0025 reported in the main manuscript are indicated with *******):

- 1. **Feature-to-feature correlational outcomes**
- SRO-Age of Acquisition - Education (*r_90_* = -0.230, *p* = 0.029);
- SRO-Graphemes Count - Education (*r_90_* = -0.344, *p* = 0.001) ***;
- SRO-Phonological Complexity - Education (*r_90_* = 0.256, *p* = 0.015);
- SRO-In-List Levenshtein - Education (*r_90_* = -0.263, *p* = 0.012);
- SRO-Dictionary Levenshtein - Education (*r_90_* = 0.234, *p* = 0.026);
- SRO-Frequency - Mini-Mental State Examination (*rho_90_* = -0.250, *p* = 0.017);
- SRO-Prevalence - Mini-Mental State Examination (*rho_90_* = -0.258, *p* = 0.014);
- SRO-Age of Acquisition (females > males; *t_88_* = -2.875, *p* = 0.005)
- SRO-Consonant/Vowel Quantity Ratio (males > females; *t_88_* = 2.628, *p* = 0.010)
  1. **Graph theory-informed outcomes**
- SRO Global Efficiency - Mini-Mental State Examination (*rho_90_* = 0.321, *p* = 0.002) ***;
- SRO Betweenness Centrality - Mini-Mental State Examination (*rho_90_* = 0.217, *p* = 0.040);
- SRO Degree – Mini-Mental State Examination (*rho_90_* = 0.323, *p* = 0.002) ***;

**References**

[1] van Heuven WJ, Mandera P, Keuleers E, Brysbaert M. (2014) SUBTLEX-UK: a new and improved word frequency database for British English. *Q. J. Exp. Psychol.* 67, 1176-1190.

**Figure S1**


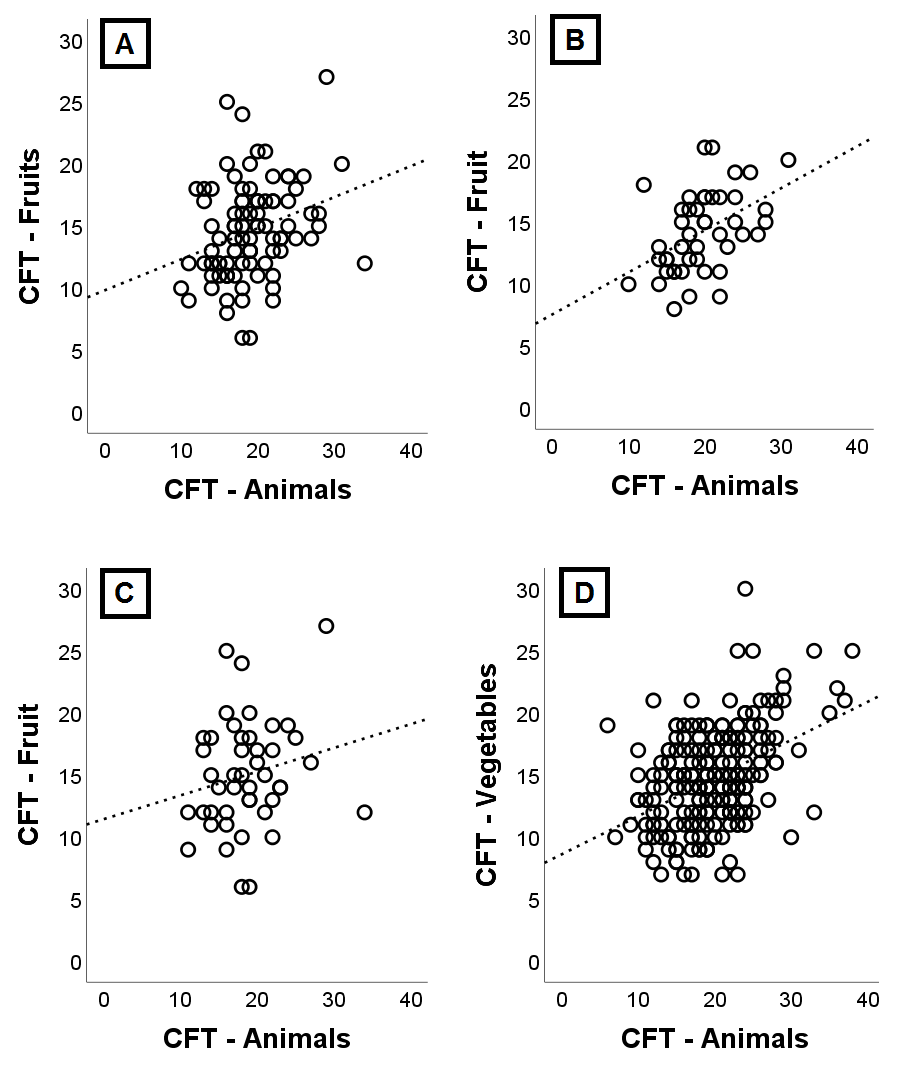


**Figure S1**. CFT cross-category consistency calculated: (A) in the entire cohort, *n* = 90; (B) in the group of younger adults, *n* = 45; and (C) in the group of older adults, *n* = 45. Validation in this latter age group was carried out via analysis of ≥ 70 year-old adults recruited as part of the ADNI-1 initiative (*n* = 220, categories; ‘animals’ and ‘vegetables’).
